# Supplementary material for: Identification of photoperiod-induced specific miRNAs in the adrenal glands of Sunite sheep (Ovis aries)
Source: Front Vet Sci. 2022 Jul 22;9:888207. doi: 10.3389/fvets.2022.888207 (PMC9354845; doi:10.3389/fvets.2022.888207)
Supplement: Supplementary file 1 [file Data_Sheet_1.ZIP › Supplementary file/miRDeep2 file.zip!/miRDeep2 file/Know_Structure/SP42A3/oar-mir-133.pdf]

miRBase precursor : oar-mir-133
Total read count : 340
oar-miR-133 read count : 325
remaining reads : 15

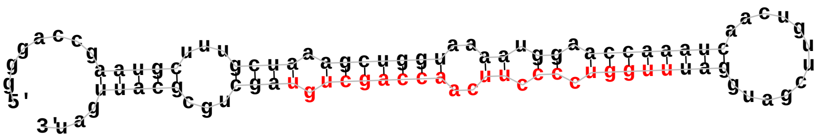

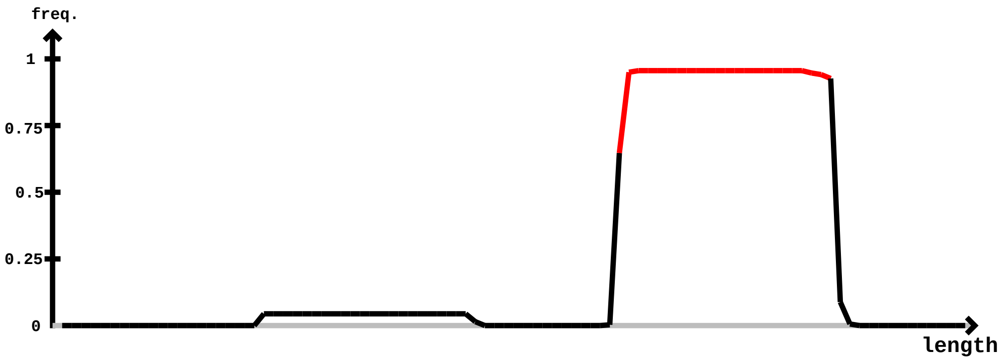

oar-miR-133

```
5'- gggaccgaaugcuuugcuaaagcugguaaaauggaaccaaaucaacuguucgauggauuugguccccuucaaccagcuguagcugcgcauugau   -3'    exp
    .......(((((...((((.(((((((..((.((.((((((((............)))))))).)).))..))))))).))))...)))))...    reads      mm        sample
    ....................agcugguaaaauggaaccaaau....................................................    10         0         seq
    ....................agcugguaaaauggaaccaaauc...................................................    5          0         seq
    ........................................................auuugguccccuucaaccagcug...............    1          0         seq
    .........................................................uuugguccccuucaaccagc.................    3          0         seq
    .........................................................uuugguccccuucaaccagcu................    2          0         seq
    .........................................................uuugguccccuucaaccagcug...............    3          0         seq
    .........................................................uuugguccccuucaaccagcugu..............    197        0         seq
    .........................................................uuugguccccuucaaccagcugua.............    14         0         seq
    ..........................................................uugguccccuucaaccagcuA...............    1          1         seq
    ..........................................................uugguccccuucaaccagcugu..............    87         0         seq
    ..........................................................uugguccccuucaaccagcugua.............    13         0         seq
    ..........................................................uugguccccuucaaccagcuguag............    2          0         seq
    ...........................................................ugguccccuucaaccagcugu..............    1          0         seq
    ...........................................................ugguccccuucaaccagcugua.............    1          0         seq
```
